# Barriers and Facilitators in Pricing and Funding Policies of European Countries That Impact the Use of Point-of-Care Diagnostics for Acute Respiratory Tract Infections in Outpatient Practices

**DOI:** 10.3390/diagnostics13233596

**Published:** 2023-12-04

**Authors:** Caroline Steigenberger, Friederike Windisch, Sabine Vogler

**Affiliations:** 1 WHO Collaborating Centre for Pharmaceutical Pricing and Reimbursement Policies, Pharmacoeconomics Department, Gesundheit Österreich GmbH (Austrian National Public Health Institute/GÖG), 1010 Vienna, Austriasabine.vogler@goeg.at (S.V.); 2 Department of Public Health, Health Services Research and Health Technology Assessment, UMIT TIROL—University for Health Sciences and Health Technology, 6060 Hall in Tirol, Austria; 3 Department of Management, Institute for Public Management and Governance, Vienna University of Economics and Business, 1020 Vienna, Austria; 4 Department of Health Care Management, Technical University of Berlin, 10623 Berlin, Germany

**Keywords:** diagnostic techniques, respiratory system, respiratory tract infections, ambulatory care, drug resistance, microbial, prospective payment system, health policy

## Abstract

Antimicrobial resistance is a major global health threat, which is increased by the irrational use of antibiotics, for example, in the treatment of respiratory tract infections in community care. By using rapid point-of-care diagnostics, overuse can be avoided. However, the diagnostic tests are rarely used in most European countries. We mapped potential barriers and facilitators in health technology assessment (HTA), pricing, and funding policies related to the use of rapid diagnostics in patients with community-acquired acute respiratory tract infections. Expert interviews were conducted with representatives of public authorities from five European case study countries: Austria, Estonia, France, Poland, and Sweden. Barriers to the HTA process include the lack of evidence and limited transferability of methods established for medicines to diagnostics. There was no price regulation for the studied diagnostics in the case study countries, but prices were usually indirectly determined via procurement. The lack of price regulation and weak purchasing power due to regional procurement processes were mentioned as pricing-related barriers. Regarding funding, coverage (reimbursement) of the diagnostic tests and the optimized remuneration of physicians in their use were mentioned as facilitators. There is potential to strengthen peri-launch policies, as optimized policies may promote the uptake of POCT.

## 1. Introduction

Antimicrobial resistance (AMR) is a major global health problem and refers to a situation when antibiotics become ineffective for the treatment of bacterial infections [1,2]. AMR can lead to treatment failures due to the inactivity of specific antibiotics against antibiotic-resistant bacteria which may require follow-up treatments. Consequences of AMR were found to be responsible for numerous deaths [3,4,5]. Globally, it was estimated based on predictive statistical models that in 2019 there were 4.95 million deaths associated to AMR [6]. Antibiotic use and the transmission of (multi)resistant pathogens between humans, animals, and the environment, for example, by travelling and trade, cause and increase AMR [7,8]. Therefore, the World Health Organization (WHO) recommends a One Health approach to respond to AMR being a public health threat [9,10].

Studies have shown that antibiotics are often applied unnecessarily, especially for community-acquired acute respiratory tract infections (CA-ARTIs) in outpatient care [11,12,13]. CA-ARTIs affect primarily the lower or upper respiratory tract. Typical lower respiratory tract infections are bronchitis and pneumonia. Typical upper respiratory tract infections include sinusitis, otitis media, and the common cold. These infections are often overtreated with antibiotics since the symptoms of viral and bacterial respiratory infections are similar, e.g., cough, sore throat, runny nose, headache, or fever. In many cases, CA-ARTI patients receive antibiotics even though this may not be necessary because symptoms are not serious, or the infections are viral. More responsible use and prescribing of antibiotics is important to avoid or, at least, reduce the risk of future multiresistant bacterial infections which increasingly become untreatable. We define responsible prescribing of antibiotics as avoiding the use of antibiotics when there is no reasonable indication that the antibiotic may be of benefit to the patient but instead may be of disadvantage. 

To assess whether the severity of the symptoms necessitates the prescription of antibiotics, a general practitioner (GP) can apply a rapid diagnostic point-of-care test (POCT), which can be smear- or blood-based, to determine whether or not the pathogens causing the CA-ARTI are bacteria. In this paper, the terms diagnostics and tests are used interchangeably. The usefulness of rapid diagnostics in antibiotic prescribing decisions was emphasized by research conducted in a Horizon 2020 project [14]. POCT have proven to be a suitable health technology to support the appropriate prescribing of antibiotics and to reduce their unnecessary use [15]. 

POCTs have been used in the European healthcare systems for years. Nevertheless, its use by GPs across countries has been low. At the same time, the use and prescribing of antibiotics vary across European countries [5,16], which points to cases of irresponsible prescribing of antibiotics in countries with a comparable disease burden. This suggests a need for improvement in antibiotic prescribing practice [17].

The consequences of AMR will be even more critical in the future when resistance to existing antibiotics develops faster than new antibiotics can be provided, and no reserve antibiotics would be available [9]. In addition to medical consequences, AMR has a significant negative economic impact on healthcare systems [18,19,20]. Public health expenditure attributed to AMR is estimated to be comparable to the treatment costs of cancer and rheumatoid arthritis [21]. Therefore, the use of rapid POCTs in patients with CA-ARTI, conducted in GP practice, has the potential to reduce long-term costs for the healthcare system, enhance the quality of the treatment, and prevent harm to society by resistant pathogens in the future. The Innovative Medicines Initiative (IMI)-funded project VALUE-Dx [22], within which our research took place, aims to reduce AMR by changing the medical practice of POCT use in CA-ARTI patients by implementing evidence-based antibiotic prescribing in the community care setting [22]. There are some mechanisms to achieve this, including practices and policies in the peri-launch phase. This phase starts with the CE-marking which indicates that the product meets statutory requirements and comprises health technology assessment (HTA), pricing, funding, and procurement of POCTs and ends with the application of the POCTs in practice [23]. 

There was limited knowledge if and how these policies were implemented for diagnostics [24,25]. Earlier research was conducted under the VALUE-Dx project to survey and map the status of the implementation of the HTA, pricing, and funding policies for diagnostic tests, including POCTs, in European countries [23,26]. Our study followed up on the mapping of peri-launch policies that were applied to POCTs used in general practices in the field of community-acquired variants of ARTIs. Tests that are sent to laboratories or used in the hospital sector (inpatient care) were not in the scope of this study.

The objective of our study was to identify the barriers and facilitators in HTA, pricing, and funding policies in European countries with the potential to impact doctors’ application of POCTs in CA-ARTI patients in European countries. The purpose of our country case studies was to understand how POCTs were managed during the peri-launch phase in different countries and what barriers hindered the adoption of POCTs in the context of the respective healthcare system, but also which policies proved beneficial in countries with high POCT uptake.

## 2. Materials and Methods

### 2.1. Design of the Case Study

Prior research consisting of a literature review and a survey, which was previously published as a technical report under the VALUE-Dx project [26], showed that the established methods have limitations in generating or describing information on HTA, pricing, and funding policies applied for POCTs. While a written survey of competent authorities, which aimed to map these policies for POCT in European countries, offered the first descriptive findings for a cross-country comparison [26], for the follow-up study of the details of the policies, research methods were needed to allow for the investigation at a deeper level. The written survey of 17 public authorities with respect to POCT pricing and reimbursement in Europe provided important first insights into national pricing and funding policies for diagnostics [23] but also raised further questions. We thus needed a methodology which would allow us to learn the details of the design of the identified policies (“how does it work?”) and identify levers for possible impacts (“why does the policy work (and why not?”). 

Therefore, we opted for a case study approach. We considered this approach for the purpose of our research questions as the most appropriate approach to gain an understanding of potential enabling or hindering factors related to HTA, pricing, and funding policies that can be used to explain why POCTs are rarely applied in outpatient care in selected European countries.

In the preparation for the interviews, grey literature, peer-reviewed literature, and legal documents were searched and screened to gain an understanding of the processes and practices regarding the HTA, pricing, and funding policies applied to point-of-care (POC) testing in the respective countries to customize the interview guide by considering the country context. 

### 2.2. Selection of Case Study Countries

The selection of the studied European countries was guided by our aim to ensure diversity regarding country size (i.e., market size), geographic location, and organization of the healthcare system. Indicators of antibiotic consumption and the use of POCTs related to our research were also considered. 

Based on these criteria, Austria, Estonia, France, Poland, and Sweden were chosen as case study countries. Thus, the country sample includes large and small countries, decentralized and centralized healthcare systems as well as countries with different economic status (Table 1).

### 2.3. Recruitment of Interviewed Experts

Between December 2021 and May 2022, participants for the expert interviews were recruited from public authorities in the five selected countries, and for each country one semi-structured interview with one or more interviewees was conducted. It was challenging to find knowledgeable interview partners, but we took advantage of our relationship with the members of the Subgroup of Medical Devices of the Pharmaceutical Pricing and Reimbursement (PPRI) network [30]. One of the objectives of the PPRI network is to support policy makers in the field of pharmaceutical pricing and reimbursement by offering a platform for exchange. The PPRI members commit to providing and sharing data and contributing to the network. Therefore, they are used to interview requests and have contributed to surveys and interviews in the past [31,32]. Most interviewees were approached through this network.

The interviewed experts were staff (or former staff) of the Estonian Health Insurance Fund, the Ministry of Health in Poland, and the Dental and Pharmaceutical Benefits Agency (TLV) in Sweden. In France, the interview was conducted as a focus group and involved representatives of the French National Authority for Health (HAS) and the Economic Committee for Health Products (CEPS). In Austria, the interview partner (Austrian Social Health Insurance Fund/ÖGK) opted for a response in writing. 

### 2.4. Data Collection and Validation by Respondents

Semi-structured qualitative expert interviews were conducted with the respondents described above to learn about enabling and hindering factors in the HTA, pricing, and funding policy setting which have the potential to impact the use of POCTs. Informed consent was obtained by the experts prior to the interviews. Two researchers participated in the interviews: one researcher facilitated the interview, and the other took notes. Based on previous experience, health authority representatives tend to more frequently decline to be available for an interview if it is recorded. To ensure an atmosphere of trust, in which the interviewees could speak out, and to protect the interviewees due to the high level of uncertainty regarding the knowledge in the area studied which was the peri-launch policies applied to diagnostics, the interviews were not recorded. 

The role of the interviewer was on purpose more participative than usual in qualitative interviews. During the interviews, the facilitating interviewer was deliberately asking questions closely linked to the country context. The in-depth preparation of the interviewer regarding the health systems of the studied countries and the theoretical knowledge of the peri-launch policies and potential challenges proved to be beneficial for identifying potential barriers and facilitators related to the peri-launch policies that could impact the use of POCTs. 

After each interview, the minutes which summarized the information that the interviewees had shared were produced. This document also included a preliminary analysis of the barriers and facilitators for POCT uptake in the case study country (for validation purposes) which was conducted by the researchers based on the statements of the interviewees. This analysis was structured along the three main areas of interest: HTA, pricing, and funding policies for POCT used in patients with CA-ARTIs. The elements of the interview that were, for example, only related to the inpatient setting or to other disease areas, were not further considered.

The interviewees were invited to verify the correctness of the minutes drafted by the researchers and to validate the draft analysis on barriers and facilitators. Information was validated and approved by the policy experts for all case study countries. This validation process of the data material collected from the interviews was deemed crucial to ensure the highest levels of accuracy of the statements as far as possible. In the data analysis, we proceeded with mapping these barriers and facilitators validated by the experts, which could be clearly assigned to a country.

### 2.5. Data Analysis

Data from the interviews were analyzed applying a qualitative content analysis integrating a deductive–inductive approach. To accomplish this, a conceptual framework was applied to establish the main areas of interest for which the information was expected to be provided from the interviews. The analysis of each country-case study was guided by the rationale that HTA, pricing, and funding are standard activities in the peri-launch phase, as described in the work published by Vogler et al., 2022 [23], which served as a guiding conceptual framework for the deductive part of the qualitative content analysis. Therefore, policies related to the HTA, pricing, and funding of POCT were the main areas of interest for the enabling and hindering factors. The information from the interviews, which was summarized in the form of protocols, was inductively added to the main areas of interest. As common practice in qualitative data analysis, every aspect of the information was considered equally important, without any subjective evaluations. Based on the information in the minutes, the fourth thematic area was generated to comprise the remainders. These additional aspects were primarily related to the prerequisites and accompanying measures and, therefore, summarized as overarching topics.

In addition to the description of the current situation and the qualitative content analysis, which is based on the minutes of the interviews, we aimed to explore the generalizability of our findings on barriers and facilitators across countries in the identified country-specific context, which may be relevant for other countries with similar healthcare systems. Given the rather low number of the interviewed experts, we refrained from discussing the transferability of the findings from the interviews to other settings but opted for a different approach instead. Four good practice examples were selected with the purpose to showcasing comprehensive strategies that address challenges in the peri-launch phase on the superordinate level. These examples were identified during the interviews and are presumed to be of high generalizability across European countries. 

### 2.6. Review Process

To enhance the validity and quality of our findings, we implemented a comprehensive review process mainly in the VALUE-Dx consortium consisting of experts from academia, industry, and professional organizations [33] but also in the PPRI network [31]. We presented the processed interview results to the VALUE-Dx consortium, the Expert Advisory Panel of the VALUE-Dx project, and the PPRI MD subgroup in three separate meetings. Additionally, the three groups received documents with a compilation of the results via email in advance, so that they could review them prior to the meeting and use the meeting to ask questions and provide comments. Through this review process, no additional findings were added to the interviews, but the description of the findings was refined to improve the comprehensibility for people who were not involved in the interviews or were not familiar with the country settings. Potentially misleading wording was rephrased.

## 3. Results

The interviews with the experts from five different European countries (Austria, Estonia, France, Poland, and Sweden) provided information on all the areas of interest, which are HTA, funding, and pricing of POCTs, the main areas of the peri-launch phase according to the conceptual framework applied to the data analysis. The barriers and facilitators related to the use of POCTs and to the implementation of policies in the peri-launch phase were identified based on the perspective of public authorities and regulatory and policy practice. This section is structured in three parts: first, the description of the status quo regarding the policies applied in the peri-launch phase in the studied countries; second, the findings related to the barriers and facilitators of the implementation of POCTs related to the peri-launch phase; and third, a summary of good practice examples in the study countries identified in the expert interviews.

### 3.1. Peri-Launch Policies Applied in the Case Study Countries

Based on the information published in prior research, supplemented by additional information from the interviews, the current status of the peri-launch policies applied to POCTs used in CA-ARTI patients is described in Table 2. 

### 3.2. Barriers and Facilitators Related to the Peri-Launch Phase

A mapping of potential facilitating and hindering factors in the policies derived from the interviews is provided in Table 3. It should be noted that in some cases interviewees reported barriers and facilitators which may apply to some health systems but not necessarily to their own health system (e.g., ideas which were suggested but not yet piloted in the interviewee’s country). During the analysis of the barriers and facilitators related to the pricing and funding policies, which are the main focus of this research, the need became apparent to broaden these two areas and to add procurement to the pricing policies and other demand-side measures to the funding policies. More detailed explanations are provided in the following subsections. The results are presented separately for the three areas: HTA, pricing and procurement, and funding policies. In the interviews, several additional facilitators and barriers were mentioned; they are also included in Table 3 if related and of relevance to our studied policies.

The barriers and facilitators in our analysis, which are presented in Table 3, do not hinder or facilitate the uptake of POCTs by GPs directly but rather affect the effectiveness HTA, pricing, and funding policies, which ultimately contribute to the achievement of the policy objectives (e.g., POCT uptake). In addition to the barriers and facilitators related to the HTA, pricing, and funding policies, the country case studies also identified good practice examples in the case study countries, which are presented in Section 3.3.

#### 3.2.1. HTA

Only two case study countries (France and Sweden) introduced systematic benefit assessment processes for medical devices, however, not for POCTs. In principle, Swedish regions have to apply an HTA, but there is no information available whether or not an HTA has been used for POCT devices. Still, the case study countries, in particular the two countries with existing, well-defined HTA processes for other medical devices, could share some reflections on possible barriers and facilitators given their learnings from other devices.

Overall, all the experts stated the lack of good evidence to analyze in an HTA as the main challenge. The experts from France indicated a potential for collaboration with industry to receive data and emphasized a need for early stakeholder involvement. The experts from Poland and France reported challenges related to the lack of transparency and regulation with respect to the HTA process for POCT and the lack of clinical evidence for evaluating the additional benefit of applying POCT for CA-ARTIs in the outpatient setting. Studies comparing different POCTs are missing, and this hinders a sound comparative effectiveness assessment. Publicly disseminated information from HTA authorities on the expected methodology and outcomes for HTA dossiers was highlighted as a facilitator, as this could improve the availability of relevant evidence and, thus, the quality of an HTA.

Besides the evidence on the effectiveness of POCTs, the experts indicated the potential of considering the users’ perspective in clinical studies to gain an understanding of the behavioral barriers to the use of novel diagnostics by physicians. Studies could also develop clinical and economic models identifying the epidemiology and associated cost of measures against AMR (including the cost to society of future resistance to antimicrobials).

#### 3.2.2. Pricing

Free pricing is applied in all five countries of our country case studies. This means that the price of a POCT is set by the market, i.e., supply and demand, and not set by the public authorities. Procurement measures are subsumed under the area of pricing because they influence the price, for example by purchasing larger quantities of POCTs through joint procurement, which generally reduces the price per unit. The EU procurement legislation framework allows for the optimization of tendering procedures with regard to the diversification of the award criteria (additional criteria other than solely price-based criteria) and the use of flexibilities in the procurement law are highlighted as possible facilitators to be used.

Pricing and procurement are a national competence in the EU Member States, and procurement procedures are frequently implemented at the regional (e.g., Sweden) or institutional (e.g., hospitals) level. Thus, this weakens the purchasing power of public procurers which was seen as a barrier.

#### 3.2.3. Funding

With respect to funding, the main facilitators mentioned include the coverage (reimbursement) of the POCT for patients and the appropriate remuneration of physicians for providing the service (i.e., remuneration which covers the costs of medical staff, storage, and other costs related to the POCT use). The perceived insufficient funding was reported to result in the lack of willingness of GPs to use POCTs. It was stated as a barrier that there is no option for higher reimbursement for innovative POCTs, which usually have a higher price, because the third-party payer or the Ministry of Health defined a fixed remuneration tariff (in Poland and France).

#### 3.2.4. Related Overarching Topics

In addition, knowledge of physicians, treatment guidelines, prescription monitoring, awareness of patients, and AMR competence were frequently mentioned in all interviews as factors that are directly related to the use of POCTs by GPs. These topics were not within the scope of our research focus, but the importance of these factors was frequently mentioned in direct relation to the peri-launch policies, which are crucial for their success and effectiveness. In general, the potential barriers include the high degree of decentralization of relevant competences for pricing (e.g., price negotiations), funding and other policy areas, insufficient implementation of national action plans, or no consequences for overprescribing antibiotics. The potential facilitators mentioned are the inclusion of POCT use in treatment guidelines and a surveillance system that regularly reports the increases or decreases in the AMR numbers, as well as diagnosis monitoring and vigilance to keep the awareness of physicians and the general population about POC testing high.

### 3.3. Good Practice Examples

The following good practice examples were mentioned during the interviews, which could also serve as models for other countries.

#### 3.3.1. Coverage with Evidence

As a measure to encourage improvement in the quality of data submitted in the HTA applications by suppliers, the French HTA agency HAS (French National Authority for Health) has increasingly negotiated Coverage with Evidence Development (CED) agreements with suppliers of innovative devices, including diagnostics, in the recent five years. These agreements allow managing uncertainty and support granting early access despite the lack of data. More importantly, they contribute to data collection, which is part of the agreement since the final reimbursement decision at the end of the CED period is based on clinical data.

#### 3.3.2. HTA Methodology Targeted at POCTs

To support the funding decisions and to develop further the HTA methodology to account for the specificities of a POCT, a joint HTA at the EU level was conducted under the lead of the Irish HTA body HIQA [34]. Several HTA agencies from different European countries collaborated in the assessment of c-reactive protein POCTs to guide antibiotic prescribing. Methodological standards from the EUnetHTA HTA Core Model [35,36,37], which were published by the European Network for Health Technology Assessment (EUnetHTA), were applied and, where necessary, slightly adapted to be valuable for POCTs. 

In this rapid effectiveness assessment, not only the HTA methodology was developed further for diagnostics, but it also serves as a good practice example of joining forces in HTA, especially for health technologies that do not have national priority as noninvasive medical devices in general. The Austrian Social Insurance authorities published a summary report of the findings of the joint HTA report in its official national language, German, in addition to the full HTA report in English to meet national requirements. This example is considered as a good learning for a resource-efficient assessment and publication policy.

#### 3.3.3. Stakeholder Involvement Targeted at Physicians

The third good practice example is from Estonia and concerns the activities to strengthen the role of physicians in policy processes. Early involvement of physicians in policy planning and their consultation on practical issues related to POCT application facilitates realistic planning of resources and an early identification of future challenges. In addition, continuous stakeholder involvement in funding negotiations enables a realistic estimation of actual costs and enables fair reimbursement and remuneration. 

#### 3.3.4. Pricing and Funding Policies Embedded in Overall Measures against AMR

The fourth good practice example, which was mentioned by the Swedish interviewee, is to provide sufficient attention, including funding, to AMR-related measures. These do not concern the pricing and funding policies per se but include, for example, education for health professionals and society, public awareness campaigns, and funding for qualified experts in a professional medical society with the focus on infectiology. Overall, such measures and initiatives to combat AMR would contribute to raising the awareness of doctors and patients with regard to POC testing and thus improve the acceptability of POC testing in GP practices, as the Swedish initiative STRAMA has demonstrated [38].

## 4. Discussion

Barriers and facilitators for POCT use were strongly interrelated with each other and with the country setting and the healthcare system. The complex and resource-intensive HTA process was reported as a challenge for public authorities but was not considered a barrier to the use of POCTs since it is not a prerequisite for funding decisions in most countries. Moreover, an HTA was not mandatory for the launch of a diagnostic test. If a benefit assessment was to be conducted, the main challenge would be the lack of evidence and limited human resources and capacities of the responsible authorities. The main barrier to the POCT use was the lack of funding when the overall costs for the GPs were considered higher than the allocated funding, and there was no separate remuneration for the service of applying the POCT. Sufficient funding and financial incentives were mentioned as the main facilitators of the POCT use prior to prescribing antibiotics.

All four good practice examples highlight the importance of stakeholder engagement, which is also an option to address the existing lack of information by some stakeholders in the healthcare system. This challenge of the lack of transparency is also linked to another challenge, which is the fragmented payer landscape for HTAs conducted at the regional level resulting in the redundancies and duplication of efforts. In Sweden, for example, it is not always transparent whether an individual payer (e.g., a region) has already conducted an HTA and what the outcome was, and thus other regions feel the need to conduct their own HTA. Transparent publication of HTA reports, which is made available to other regions, can save resources (i.e., costs and capacity for HTA bodies).

Our survey in addition to the interviews with government officials proved to be a valuable way to identify relevant barriers to and facilitators of POC testing given the lack of published information. When interpreting the results of our interviews, it is important to consider the country context because potential barriers and facilitators, or their root causes, are usually related to the country setting and the established processes in the healthcare system. For example, in countries where GPs are financed via capitation fees, the introduction of additional funding for the diagnostic tests will require different implementation approaches compared to those used in the fee-for-service-based funding systems. 

### 4.1. Context of the Literature and Other Programs

Insufficient funding of POC testing was also reported to be a challenge for GPs in Germany and thus a potential barrier to POCT uptake. The purchase price of the diagnostic tests exceeded the reimbursed amount, and GPs reported experiencing a financial disadvantage when applying the POCT. In some regions, the Association of Statutory Health Insurance Physicians and the State Association of General Practitioners reacted by providing additional remuneration on top of the standard, nationally agreed remuneration. Thus, some (but not all) regions allocated a budget with the purpose to reduce AMR [39].

Our findings add to the systematic review on the barriers to and facilitators of patient access to new innovative medical devices [40]. Thus, this review addressed a much broader topic in terms of medical devices (in general) and policies (the whole life cycle). Overall, this systematic review also pointed to the importance of the funding and pricing policies, by identifying the reimbursement process as a crucial factor to bring new medical devices to the market, which might also be applicable to POCTs used in the outpatient sector. In addition to challenges related to the regulatory aspects, like the lack of clarity with regards to the European legislation and complex market approval procedures, there are also barriers related to the peri-launch phase. These include insufficient data collection, differing requirements for evidence across countries, complex and time-consuming regional reimbursement decisions, and individual procurement [40]. 

### 4.2. Limitations and Strengths

This study has a few limitations. It was difficult to identify knowledgeable interviewees who were able to cover all relevant peri-launch policy aspects around POC testing, and who were available for an interview. Contacting experts through the PPRI Subgroup on Medical Devices, whose members are encouraged to participate in surveys as a requirement for being a network member as described in the Materials and Methods section, was very useful but did not allow us to conduct focus group discussions with experts from different institutions in each country, since all the PPRI MD members represent competent authorities. A discussion with the representatives of various institutions within a country could have triggered a multiperspective discussion that might have indicated how the challenges within a country might be addressed. Thus, we could not avoid the variation in the methodology of the data collection (e.g., mixture of focus groups and interviews with individual people), and in four countries, we relied solely on one expert, which may limit the validity of this study results. In one country, no interview was possible, so we accepted the alternative of a written response by an expert. A higher number of interviewees and a higher number of case study countries would likely have resulted in identifying further facilitators and barriers, but no further experts were available for the interview. Another limitation is that the experts from public authorities had special focus and knowledge in one area of the studied peri-launch phase and in the related policies for diagnostics but not necessarily regarding POCT. Thus, responses, including those concerning the above-mentioned facilitators and barriers, might be overrepresented in the respective area of experience of the interviewees. At the same time, it is possible that the interviewed experts did not discuss topics that would be relevant in the respective country. We were not able to achieve saturation in terms of content, as is intended in qualitative studies. Furthermore, the triangulation of the results via a comparison with the scientific publications was not possible given the lack of the literature in our research area. To compensate for these weaknesses, we instead incorporated an extensive review process in which the experts (e.g., from the VALUE-Dx consortium and the PPRI network) were given the opportunity to review the results of our qualitative content analysis, assess them for plausibility, and provide feedback on possible missing or incorrectly identified barriers and facilitators.

Despite these limitations, this study stands out as the first of its kind for non-high-risk in vitro diagnostics, and the findings on the barriers and facilitators can form the basis for the development and optimization of practical and relevant peri-launch policies.

### 4.3. Policy Implications

Our research found that there is limited expertise on HTA-, pricing-, and funding policy-related aspects among public authorities governing medical devices, including (in vitro) diagnostics. Presumably, the reason why expertise is relatively low is that, at the time of the survey (as of 2022), only a small proportion (approximately 20%) of in vitro diagnostics required a benefit assessment, for example, in France. Similar to France, HTA does not really play a role in the peri-launch processes in other countries. In addition, specific pricing and funding policies are not or only rarely applied to POCTs used in the outpatient sector [23,26]. Our research can support policy makers in understanding the impact of the healthcare system characteristics and the inter-linkages of different areas on the success of individual policies to be able to better assess what is fit for purpose in their own country. 

Despite the value of our findings for high-income countries, we fear that the transferability of our findings to other countries is limited due to the strong impact of the country settings and healthcare systems on the challenges and opportunities that arise. We primarily focused on high-income countries with well-developed systems that should be pioneers in the implementation of POC testing in standard care and in defining process-related policies for the peri-launch phase. However, even in these countries, there are many barriers to the implementation and use of POCTs in practice. The problems addressed in our research also seem applicable to other high-income countries, with the exception of Sweden, which we also highlighted as an example of good practice. However, it appears that the transferability to low-and middle-income countries may only be feasible at a later stage upon the progress of the regulatory and policy frameworks.

Consultation with physicians from the community care setting on the topic of the uptake of POCT could also involve the discussion of current challenges and potential disincentives in remuneration. Our findings also raise the awareness of the fact that refraining from policy implementation (in the areas of pricing and funding and beyond) also comes with a cost to the healthcare system. By mapping the situation of current practice regarding the peri-launch policies in the case study countries and identifying the respective barriers to and facilitators of the use of POCT, we offer a basis for subsequent policy recommendations in the country- and healthcare system-specific context, developed within the scope of the VALUE-Dx project and planned to be published in a follow-up publication.

## 5. Conclusions

The identified barriers and facilitators in the peri-launch phase for POCT uptake are diverse but, in most cases, strongly related to the country context of the respective healthcare system. Currently, free pricing is applied in all investigated country case studies, and there are large differences in the funding for the application of POC testing in the community care setting, consisting of the reimbursement for the costs of the POCT and the remuneration for the service of the application of the test. Optimizing the funding offers the potential to increase the uptake of POCTs. It is important for policy makers to recognize that the nonadoption of the peri-launch policies may also impose costs on the healthcare system in the long term and may leave the potentials of pricing and funding untapped. Policy-related measures in the peri-launch phase should not be assessed independently of other measures against AMR, because different measures interact and can impact each other. For example, overarching measures such as a national action plan against antimicrobial resistance increase the acceptance of POC testing in the country and might influence the uptake of POCTs indirectly if the barriers related to funding are removed.

## Figures and Tables

**Table 1:** Characteristics of case study countries (2021 values).

Country	Population (in Thousands) ^1^	Characteristics of Health System and Funding	HE in Current USD per Capita ^1,2^	HE in % of GDP ^1^	Antibiotic Use (DDD per 1000) ^1,2^	Use of Diagnostics to Detect Antibiotic Susceptibility ^3^
Austria	8933	Decentralized, contribution-based social health insurance funded by contributions from employees, employers, and the government	6491	12	7.19	50%; limited recommendation for use in clinical guidelines; no information available regarding the POCT use in CA-ARTI patients in practice
Estonia	1330	Centralized, tax-funded Beveridge system with national health insurance	2036	7	8.65	68%; application of diagnostics recommended for all relevant infections; POCTs are commonly used for CA-ARTI patients in practice
France	67,320	Centralized, tax-funded Beveridge system with national health insurance	4769	12	19.31	44%; application of diagnostics recommended for all relevant infections; POCTs are rarely used for CA-ARTI patients in practice
Poland	37,840	Centralized national social health insurance system, funded by contributions from employees, employers, and the government	1183	7	18.80	40%; limited recommendation for use in clinical guidelines; POCTs are primarily used in CA-ARTI patients in private GP practices, which means that they are financed by the patients
Sweden	10,379	Decentralized tax-funded Beveridge system	6915	11	8.65	61%; application of diagnostics recommended for all relevant infections; POCTs are commonly used in CA-ARTI patients in practice

**Abbreviations:** CA-ARTIs: community-acquired acute respiratory tract infections; DDD: defined daily dose, which is the assumed average maintenance dose per day for a drug used for its main indication in adults; GDP: gross domestic product; HE: health expenditure; POCT: point-of-care test. ^1^ Population figures and figures related to health expenditure are taken from the Global Health Expenditure Database 2021 [27]. ^2^ Antibiotic use in DDD per 1000 inhabitants per day. Antibiotic use figures refer to the ATC groups: J01A, J01c, J01D, J01E, J01F, J01M, J01X in community care, expressed as DDD per 1000 inhabitants per day in 2021, as reported by the European Centre for Disease Prevention and Control [28]. The EU/EEA average is 14.88 DDD per 1000 inhabitants per day and refers to the population-weighted mean consumption based on reported or imputed antimicrobial consumption data from 29 EU/EEA countries. ^3^ Proportions of POCT use refer to the respondents (all diagnoses) who have taken antibiotics in the last 12 months (*N* = 8416) based on the report on a survey commissioned by the European Commission [29]. The current use of diagnostics to detect antibiotic susceptibility is characterized as “limited” if it is only recommended for selected infections or not routinely used.

**Table 2:** Peri-launch policies applied to POCT in the studied countries.

Country	HTA	Pricing	Funding
Austria	No systematic use of HTAs as part of the decision-making process for P + F. An HTA is conducted for selected topics (mostly high-risk products (IIb and III)) but not for Dx and POCT.	Free pricing	Publicly funded through the reimbursement of the POCT cost and remuneration for the doctor’s service of applying the POCT. Differences may arise between the social insurance funds.
Estonia	No systematic use of HTAs as part of the decision-making process for P + F. An HTA is conducted for selected topics but not for Dx and POCT.	Free pricing, indirect price control through public procurement	Publicly funded through the reimbursement of the POCT cost and remuneration for the doctor’s service of applying the POCT. Reimbursement tariffs are annually updated based on the information provided by and requested from the manufacturer. If suppliers consider the reimbursement tariffs too low, they can contact the social insurance provider and negotiate an update of the tariffs.
France	Systematic use of HTAs as part of the decision-making process for P + F for defined MDs but not for Dx and for POCT devices. At the time of the survey, an assessment committee dedicated to diagnostics was being established in the HTA body.	Free pricing	Publicly funded through the remuneration for the doctor’s service of applying the POCT. Patient co-payments may be required for the POCT. In practice, co-payment is covered by a “mutual” (complementary) health insurance which most French citizens have.
Poland	No HTA conducted for pricing and funding decisions for Dx and POCT.	Free pricing	Publicly funded through the remuneration for the doctor’s service of applying the POCTs; co-payments for the POCT may be required from the patient.
Sweden	Systematic use of HTAs as part of the decision-making process for P + F for three defined groups of MDs (for stoma care, administration of medicines, and the measurement of pharmaceutical levels). For other MDs, including POCTs, the regions (payers) can conduct an HTA or requested one from the national authority TLV.	Free pricing, indirect price control through public procurement	Full cost-coverage. Publicly funded, as POCTs devices are procured by the regions and provided for free to doctors *.

* This finding was reported differently in prior research [26], as no-cost coverage, but our expert from Sweden confirmed that the costs for POCT are fully covered by the healthcare system. **Abbreviations**: Dx: diagnostics; HTA: health technology assessment; MD: medical device; P + F: pricing and funding; POCT: point-of-care test; TLV: the Swedish Dental and Pharmaceutical Benefits Agency. **HTA** refers to the process of evaluating the clinical effectiveness, cost-effectiveness, and potential ethical and social implications of health technologies, including diagnostics. **Pricing** for diagnostics refers to whether or not there is regulation on the pricing of diagnostic tests, such as price caps or negotiation with manufacturers. **Funding** for diagnostics refers to the sources of funding for diagnostic tests, such as public funding or private insurance. The policies described are based on the expert interviews and may not be comprehensive or up to date.

**Table 3:** Barriers and facilitators in policies related to HTA, pricing, and funding.

Topic	Barriers	Facilitators
**HTA**		
Quality of data	No or limited evidence to inform an HTA (France, Poland). Limited data/proof of patient benefit (France).	Contractual arrangements between public authorities and suppliers (managed-entry agreements, e.g., coverage with evidence development) which link the (final) funding decision of the public payer to the clinical data and thus encourage data collection (France).
Developing a methodology for HTA which is appropriate for CA-ARTI POCTs	Methodological challenges, in particular, in the assessment of patient benefit (France).	Further development of the methodology, which considers the perspective of the users (physicians) as well as particularities for POCTs (idea, suggested by France and Poland)
Legislative basis	No legislation mandating the conduct and use of HTA within the decision-making process (Poland).	New EU MD regulation will push manufacturers and suppliers towards clinical studies, which would then also be available in follow-up processes such as HTA (France).
Organization of the P + F system	No product-specific reimbursement process; thus, no need for an HTA process is perceived (France, Poland). Fragmented payer landscape as a result of conducting HTAs for regional jurisdictions and not nationally (Sweden).	-
Costs and capacity (from the perspective of HTA bodies)	Conducting a full HTA is considered as too expensive (Estonia). HTA bodies across Europe may lack the capacity to conduct HTAs for POCT (France).	-
Costs and capacity (from the supplier’s perspective)	Limited interest and expertise of suppliers to produce data needed for an HTA (France)	HTA agencies should encourage and support through capacity building in generating clinical data (France). HTA agencies should offer early scientific advice to manufacturers (France). Diagnostic manufacturers could liaise with pharmaceutical manufacturers (e.g., for companion diagnostics), who are more experienced in data collection, to allow for cross-learning (idea, raised by France).
Priority setting	Policy makers are less focused on diagnostics compared to medicines (Poland)	Workplan, possibly linked to an AMR roadmap, requesting the HTA body to focus on the HTAs of POCT (France).
**Pricing and procurement**		
Organization of the pricing and procurement system	Fragmented procurer landscape with individual tenders may lead to differences in the availability of POCTs across the regions (Sweden) and untapped potential for collaboration (Sweden).	Possibility to negotiate prices at the national level (even when there are multiple payers) strengthens the pricing process and capacity (Sweden). Creation of a common understanding that disregarding pricing and procurement policies has a negative financial impact (Austria).
European framework	Pricing as a national competence in the EU weakens the pricing process (Sweden).	-
Pricing in the supply chain	-	A legislative and policy framework which considers all price components, such as price regulation (e.g., margin regulation), targeted at actors in the supply chain (e.g., wholesalers) (Poland).
Affordable prices	No perceived need for price regulation and subsequent pricing policies for POCT given their comparably low prices (Estonia) *.	-
Market structure	-	“Healthy market” with a sufficient number of suppliers, which allows for competition and assured availability (Poland)
Procurement procedures	Tedious tender procedures can be challenging and time-intensive (Austria).	Tender specifications with quality criteria such as antibiotic susceptibility as an award criterion (Poland). Design of national procurement contracts which apply a cap in line with affordability of the system (Estonia, Poland).
**Funding**		
Remuneration for the service of using POCT	Remuneration of doctors is solely based on a capitation fee, without any fee-for-service remuneration; costs for procuring POCTs by the doctors are not covered by the fee-for-service remuneration (France). Costs for establishing and maintaining the infrastructure for offering POC testing in general practice (e.g., equipment, staff, storage) may not be fully covered (Poland). Due to national regulations, in Sweden the distribution of POCTs is only allowed by laboratories, which must be financed (Sweden).	A funding setting in which POC testing is fully covered and physician time to apply the POCT is funded, e.g., in the GP’s salary (Estonia, Sweden).
Funding of POCT testing	Insufficient public funding for POC testing, which is not reimbursed on a product basis by the public payer; patients must pay for the test, inequities across GP practices (Poland). Suppliers consider tariffs for product-specific reimbursement insufficient to incentivize the development of innovative POCTs (France).	Having in place product-specific reimbursement mechanisms for POCT (Estonia, suggested by France and Poland). A funding system which is based on a well-designed, clear process, considering HTA findings and involving stakeholders where appropriate (suggested by Austria and France).
Flexibility allowing updates	-	Systematic procedures, with dialogue with manufacturers on cost development, to allow for regular (annual) updates (Estonia).
Implications of nonuse	No (financial) sanctions for doctors who do not use POCTs. However, there are potentially higher costs to the system to implement sanctions and there is insufficient compensation for doctors who use it (Poland).	-
Funding for antibiotics/CA-ARTI treatment	--	An integrative system in which funding for the CA-ARTI treatment (e.g., prescription of an antibiotic) is dependent on the use of a POCT prior to prescribing; a variant could be some bonus payments to doctors for responsible antibiotic prescribing (applied in Sweden).
Funding of other measures against AMR	Limited funding for measures against AMR (e.g., awareness-raising activities) (Poland, Sweden).	Successful long-term programs with sufficient funding for many years to enable high impact and quality of measures (Poland, Sweden).
**Overarching topics**		
Knowledge of physicians	Limited knowledge of physicians about AMR, antibiotics, and POC testing (France, Poland) and possibly also limited interest of physicians in the area of AMR, which reaches from diagnosis to treatment (Poland).	Educational activities targeted at doctors to improve their knowledge about POC testing for prescribing antibiotics as a supportive measure not linked to funding and pricing (Estonia, France, Poland, Sweden), coupled with a requirement by the social insurance provider to use POCTs prior to prescribing antibiotics (suggested by Poland). A culture of awareness about AMR and POC testing would be beneficial (Austria).
Treatment guidelines	-	POCT use recommended or mandated by clinical guidelines, as a supportive measure (Estonia, Poland, suggested by Austria).
Prescription monitoring	The lack of reporting back on prescription behavior, also in comparison to peers (Poland).	Monitoring of the prescription pattern of physicians, with regular reporting back to them, also with benchmarking information on the prescription behavior of other prescribers, to be combined with financial incentives for responsible prescribing of antibiotics (Sweden).
Awareness of patients	-	Awareness-raising campaigns targeted at the public to improve the general knowledge on AMR, antibiotics, and POC testing and to support the use of POCT.
AMR competence	The lack of an overall responsible institution for the cross-cutting topic of AMR at the national level (Poland).	Clarity on the responsibility for the topic of AMR in a country (Sweden).

* This item is listed as a “barrier” because while low prices help to increase the POCT usage, the affordability of prices impacts the implementation of the pricing policies due to the lack of need as the system is functioning. **Abbreviations**: AMR: antimicrobial resistance; CA-ARTIs: community-acquired acute respiratory tract infections; HTA: health technology assessment; MD: medical device; POC testing: point-of-care testing; POCT: point-of-care test.

## Data Availability

No new data besides the published information were created.
